# Isolation and Characterization of Multidrug-Resistant *Escherichia coli* and *Salmonella* spp. from Healthy and Diseased Turkeys

**DOI:** 10.3390/antibiotics9110770

**Published:** 2020-11-02

**Authors:** Md. Tawyabur, Md. Saiful Islam, Md. Abdus Sobur, Md. Jannat Hossain, Md. Muket Mahmud, Sumon Paul, Muhammad Tofazzal Hossain, Hossam M. Ashour, Md. Tanvir Rahman

**Affiliations:** 1 Department of Microbiology and Hygiene, Faculty of Veterinary Science, Bangladesh Agricultural University, Mymensingh 2202, Bangladesh; soukhin1993@gmail.com (M.T.); dvm41257@bau.edu.bd (M.S.I.); dvm38397@bau.edu.bd (M.A.S.); dvm38553@bau.edu.bd (M.J.H.); dvm41187@bau.edu.bd (M.M.M.); sumonmbjust@gmail.com (S.P.); tofazzalmh@bau.edu.bd (M.T.H.); 2 Department of Microbiology and Public Health, Khulna Agricultural University, Khulna 9208, Bangladesh; 3 Department of Integrative Biology, College of Arts and Sciences, University of South Florida, St. Petersburg, FL 33701, USA; 4 Department of Microbiology and Immunology, Faculty of Pharmacy, Cairo University, Cairo 11562, Egypt

**Keywords:** avian colibacillosis, salmonellosis, antibiotic resistance, MDR, *tetA*, public health

## Abstract

Diseases caused by *Escherichia coli* (*E. coli*) and *Salmonella* spp. can negatively impact turkey farming. The aim of this study was to isolate and characterize multidrug-resistant (MDR) *E. coli* and *Salmonella* spp. in healthy and diseased turkeys. A total of 30 fecal samples from healthy turkeys and 25 intestinal samples from diseased turkeys that died of enteritis were collected. Bacterial isolation and identification were based on biochemical properties and polymerase chain reaction (PCR). Antibiogram profiles were determined by disk diffusion. The tetracycline-resistance gene *tetA* was detected by PCR. All samples were positive for *E. coli*. Only 11 samples (11/30; 36.67%) were positive for *Salmonella* spp. from healthy turkeys, whereas 16 (16/25; 64%) samples were positive for *Salmonella* spp. from diseased turkeys. *E. coli* isolated from diseased turkeys showed higher resistance to levofloxacin, gentamicin, chloramphenicol, ciprofloxacin, streptomycin, and tetracycline. *Salmonella* spp. isolated from healthy turkeys exhibited higher resistance to gentamicin, chloramphenicol, ciprofloxacin, streptomycin, imipenem, and meropenem. All *E. coli* and *Salmonella* spp. from both healthy and diseased turkeys were resistant to erythromycin. *Salmonella* spp. from both healthy and diseased turkeys were resistant to tetracycline. Multidrug resistance was observed in both *E. coli* and *Salmonella* spp. from diseased turkeys. Finally, the *tetA* gene was detected in 93.1% of the *E. coli* isolates and in 92.59% of the *Salmonella* spp. isolates. To the best of our knowledge, this is the first study to isolate and characterize *tetA*-gene-containing MDR *E. coli* and *Salmonella* spp. from healthy and diseased turkeys in Bangladesh. Both microorganisms are of zoonotic significance and represent a significant public health challenge.

## 1. Introduction

Turkey (*Meleagris gallopavo*) farming is a profitable business in many countries. In Bangladesh, turkey farming generates a higher profit than broiler and layer farming due to lower feeding cost, higher market price, and high demand from consumers. In addition, turkey is generally more adaptable under different weather conditions and less prone to disease than other poultry birds [1,2]. In Bangladesh, there are more than 600 small- and medium-sized commercial turkey farms [3]. With strong support of the Bangladesh government, the number of farms is increasing [3]. According to the Household Income and Expenditure Survey 2016 in Bangladesh [4], the average daily protein intake per capita was 63.50 g, of which meat, poultry, and eggs contributed 12.65% of the total proteins. Furthermore, poultry contributed 37% of the overall meat production in Bangladesh [5]. In rural areas, rearing poultry is a common additional source of income [6]. The challenges of turkey farming include potential outbreaks of infectious and non-infectious diseases, which have been shown to impact more than a third of turkey farmers in Bangladesh [7]. Infections caused by *Escherichia coli* and *Salmonella* spp. have negative impacts on turkey farming as they lower egg production, reduce hatchability, and increase mortality rates [8]. Thus, the control of *E. coli* and *Salmonella* infections in turkey farms is crucial.

*E. coli* is a zoonotic commensal pathogen that is capable of causing infections in the gastrointestinal tract (GIT), respiratory tract, and bloodstream in both humans and animals [9,10]. Avian colibacillosis caused by *E. coli* is responsible for turkey cellulitis, colisepticemia, swollen head syndrome, synovitis, salpingitis, coligranuloma, osteomyelitis, omphalitis, peritonitis, panophthalmitis, and is often deadly for turkeys [11,12]. It also causes urinary tract infections (UTIs), abdominal sepsis, and meningitis. It is important to note that *E. coli* is responsible for about 80% of UTIs in humans [13,14].

*Salmonella* spp. can cause salmonellosis (especially pullorum disease and fowl typhoid) in turkeys [15,16]. *Salmonella* infections reduce hatchability, fertility, growth, and increase mortality rates in poultry [17]. Due to their zoonotic nature, *Salmonella* spp. can be transmitted to humans through the food chain. This can lead to the development of salmonellosis, gastroenteritis, enteric fever [18,19], and can sometimes cause life-threatening consequences [20].

The excessive use of antibiotics in farms led to the emergence of antibiotic-resistant bacteria such as *E. coli*, *Salmonella* spp., and *Campylobacter* spp. in poultry [21,22]. High levels of antibiotic-resistant or multidrug-resistant (MDR) *E. coli* and *Salmonella* spp. can constitute a more significant problem in turkeys than in other livestock species [21,23]. Mutations in *E. coli* and *Salmonella* spp. could result in the acquisition of antibiotic resistance [24]. Mobile genetic elements allowed bacteria to acquire and disseminate antibiotic resistance [25]. The implications of this acquired antibiotic resistance for public health necessitates attention from both clinical and economic experts [26].

Antimicrobial resistance (AMR) poses a significant threat to human health [27]. AMR is responsible for approximately 700,000 human deaths every year throughout the world [28]. This figure could significantly increase in the near future if we do not discover novel and effective antibiotics [29]. The antibiotic resistance in farm animals is clearly intertwined with the presence of this problem in humans [30,31]. In addition, the indiscriminate use of antibiotics in livestock is one of the main causes of AMR [25,26]. The overuse of antibiotics by farm owners in poultry farms, a common practice in developing countries, is a major reason for the development of MDR bacteria [32,33]. This overuse typically occurs without consulting any veterinarians and without any previous testing of the animals. The development of MDR bacteria in poultry has been previously reported in previous studies [22,33,34,35]. Poultry farmers have been using different types of poultry in recent years including broilers, layers, and turkeys. These animals are hosted close to each other, which can lead to the horizontal transmission of MDR bacteria to turkeys. The dissemination of MDR bacteria to humans exposes the population to risk, especially the immunocompromised individuals, and exacerbates healthcare costs, and ultimately increases the usage of antibiotics [36].

The present study was designed to isolate and characterize MDR *E. coli* and *Salmonella* spp. from both healthy and diseased turkeys. There is an urgent need to design proper surveillance and control programs for the detection and control of antibiotic-resistant bacteria in turkey farms.

## 2. Results

### 2.1. Prevalence of E. coli and Salmonella spp.

All 55 samples were positive for *E. coli* (using PCR targeting the *malB* gene), whereas 27 samples (27/55; 49.09%) were positive for *Salmonella* spp. (using PCR targeting the *invA* gene). The prevalence of *E. coli* in turkeys was significantly higher than *Salmonella* spp. (chi-square test, 95% CI, *p* < 0.001). The prevalence of *Salmonella* spp. was significantly higher in diseased (64%; 16/25) than in healthy turkeys (36.67; 11/30) (chi-square test, 95% CI, *p* < 0.05). No significant difference between healthy and diseased turkeys was observed in the case of *E. coli* (Table 1).

### 2.2. Antibiotic Profiles of Isolated E. coli and Salmonella spp.

Antibiotic sensitivity tests revealed that all *E. coli* isolates were resistant to erythromycin; whereas all *Salmonella* isolates were resistant to erythromycin and tetracycline. Additionally, *E. coli* isolates were resistant to ciprofloxacin (67.27%), meropenem (72.73%), and tetracycline (52.73%). *Salmonella* spp. were resistant to ciprofloxacin (44.44%) and meropenem (40.74%). *E. coli* isolates were highly sensitive to imipenem (92.73%)

*E. coli* isolated from diseased turkeys showed higher resistance to levofloxacin (chi-square test, 95% CI, *p* = 0.011), gentamicin (*p* < 0.001), chloramphenicol (*p* < 0.001), and tetracycline (*p* < 0.001); whereas isolates from healthy turkeys showed higher resistance to meropenem (*p* < 0.001). Interestingly, *Salmonella* spp. isolated from healthy turkeys exhibited higher resistance to gentamicin, chloramphenicol, ciprofloxacin, streptomycin, imipenem, and meropenem than *Salmonella* spp. isolated from diseased turkeys. However, only a few cases were statistically significant (Table 1).

### 2.3. Detection of tetA Gene

**Figure 1:**
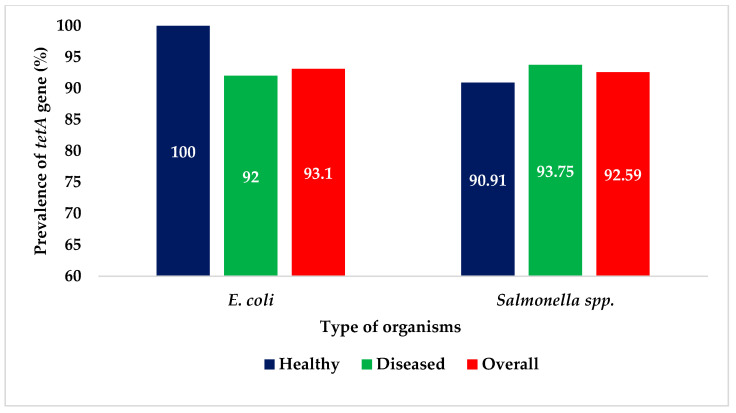
Prevalence of *tetA* gene in *E. coli* and *Salmonella* spp. isolated from turkeys.

Of the 29 *E. coli* isolates phenotypically resistant to tetracycline, *tetA* was detected in 27 (27/29; 93.1%). In the case of *Salmonella* spp., *tetA* was detected in 25 of the 27 isolates (25/27; 92.59%). The prevalence of *tetA* was similar in healthy and diseased turkeys for both *E. coli* and *Salmonella* spp. (Figure 1).

### 2.4. Detection of MDR E. coli and Salmonella spp.

As shown in Table 2, antibiogram typing revealed that most *E. coli* isolates (48/55; 87.27%) and most *Salmonella* isolates (24/27; 88.89%) exhibited multi-drug resistance. For *E. coli*, the percentage of MDR isolates was higher from diseased turkeys (24/25; 96%) than from healthy turkeys (24/30; 80%). For *Salmonella*, the percentage of MDR isolates was also higher in diseased turkeys (16/16; 100%) than in healthy turkeys (11/16; 72.72%). However, the differences were not statistically significant in either case (chi-square test, 95% CI, *p* > 0.05).

*E. coli* isolated from healthy turkeys showed eight resistance patterns, while *E. coli* isolated from diseased turkeys showed ten resistance patterns. *Salmonella* isolated from healthy and diseased turkeys showed four and seven resistance patterns, respectively (Table 2). Among the antibiogram types, pattern E-MEM-CIP showed the highest prevalence in *E. coli* (14 isolates). On the other hand, the E-CIP-TE pattern showed the highest prevalence in *Salmonella* (five isolates) (Table 2).

## 3. Discussion

In this study, we report the detection of MDR *E. coli* and *Salmonella* spp. from healthy and diseased turkeys. This is significant to human health due to the zoonotic nature of these pathogens. Moreover, most *E. coli* and *Salmonella* spp. isolates were found to be MDR, which makes it difficult to treat the infected turkeys [37,38,39,40,41,42]. Antibiograms can guide the choice of therapies for colibacillosis and salmonellosis in turkeys. The incorrect choice of antibiotics is not only associated with the development of AMR but can also have significant negative economic impacts.

Whereas all samples were positive for *E. coli*, only 49.09% (27/55) of the samples were positive for *Salmonella* spp., which were significantly more prevalent in diseased than in healthy turkeys. The isolation and characterization of *E. coli* and *Salmonella* spp. from turkeys revealed the presence of the *tetA* gene. The gut microflora of poultry typically includes *E. coli* and *Salmonella* spp. [43]. Detection of *Salmonella* spp. in diseased turkeys that died of enteritis suggests that *Salmonella* was the causative factor of enteritis. Previously, Kar et al. [8] reported the detection of *E. coli* and *Salmonella* spp. from cloacal swabs of turkeys but did not use any molecular techniques, such as the PCR technology used in this study. PCR is a robust and rapid detection method with increased sensitivity and specificity for detecting *Salmonella* in food, environmental, and clinical samples [44]. The *invA* gene has been the target for many PCR protocols, as it is found in almost all known serovars of *Salmonella* [45]. This gene encodes an inner membrane protein necessary for invasion of epithelial cells by *Salmonella* [46]. We were able to observe higher rates of *E. coli* and *Salmonella* spp. compared to the study of Kar et al. [8], which may be attributed to the highly sensitive nature of the molecular techniques used in this study.

The detection of *E. coli* and *Salmonella* spp. from fecal materials and intestinal contents of healthy turkeys indicates intestinal colonization [47]. The findings also indicate that fecal materials may be a source of transmission of *E. coli* and *Salmonella* spp. to other birds. The detection of the virulence gene *invA* in the isolated *Salmonella* spp. indicates the potential pathogenic nature of these isolates. It is also possible for these pathogens to be introduced into the food chain causing food-borne diseases [48].

Antibiotic resistance is a major public health problem. The misuse and abuse of antimicrobial agents contributed to the emergence and dissemination of antibiotic-resistant pathogens in animals and humans [49]. Location-specific information on antibiotic resistance patterns in different geographical areas is important for the successful treatment of outbreaks and infections. The isolated *E. coli* and *Salmonella* spp. were found to be resistant to levofloxacin, erythromycin, ciprofloxacin, meropenem, and tetracycline. This antibiotic resistance profile can be due to the frequent use of antibiotics in poultry for therapeutic and growth promotion purposes [32,33]. The presence of antibiotic-resistant *E. coli* and *Salmonella* spp. in fecal materials of healthy turkeys indicates the role of these birds as spreaders of resistant microorganisms in farm environments.

Several studies detected the *tetA* gene in *E. coli* and *Salmonella* spp. from dairy farms, boiler farms, house flies, and aquatic environments [31,33,50,51,52]. However, there were no studies on the detection of the *tetA* gene in *E. coli* and *Salmonella* from turkeys. Among the isolates phenotypically resistant to tetracycline, 93.1% of the *E. coli* isolates and 92.59% of *Salmonella* spp. isolates were positive for the *tetA* gene. The *tetA* has been shown to be the most common genetic component in tetracycline-resistant *E. coli* and *Salmonella* spp. [9,53,54,55]. Generally remaining in mobile genetic components (integrons, transposons, and plasmids), *tetA* can be easily transferred to different bacteria.

Resistance to carbapenems (imipenem and meropenem) may be due to the transmission of bacteria from human sources, especially that carbapenems are not approved for use in livestock [56]. Future detailed studies at the genetic level are needed to test this hypothesis. According to the WHO, carbapenem-resistant *E. coli* and *Salmonella* spp. are considered to be among the most critical pathogens [57]. The detection of carbapenem-resistant *E. coli* and *Salmonella* spp. in turkeys has to be treated as an urgent public health problem.

Antibiotic treatment failures in poultry has been highly attributed to the MDR nature of the pathogens [58]. In the present study, the majority of the isolated *E. coli* (48/55; 87.27%) and *Salmonella* spp. (24/27; 88.89%) were MDR. More MDR *E. coli* and *Salmonella* spp. were retrieved from diseased turkeys than from healthy turkeys. The higher MDR in diseased turkeys may have been caused by the selection pressure resulting from the excessive use of several classes of antibiotics. However, the differences were statistically insignificant as in Table 2 (*p* = 0.112 and *p* = 0.056 for *E. coli* and *Salmonella* spp., respectively). The statistical insignificance indicates that the bacteria were MDR regardless of whether the source was healthy or diseased turkeys. To avoid the development of MDR, the use of antibiotics should be more strategic and selective.

## 4. Materials and Methods

### 4.1. Ethics Statement

No ethical permission was required for the study. During sample collection, verbal permission was taken from farm owners.

### 4.2. Study Design

A pilot survey was conducted prior to the start of the current study to identify the different turkey farming areas in Bangladesh, disease outbreaks in these farms, and antibiotic treatment regimens. Based on the survey results, seven antibiotics were selected. In addition, two carbapenem antibiotics were included based on reports that indicated that *E. coli* could be resistant to carbapenems in poultry [31,50,59]. Guided by bird mortality rates and antibiotic use reports from the survey, five farms from two districts were selected for sample collection. The birds were categorized into healthy and diseased birds. Six healthy and five diseased bird samples were randomly collected from each farm resulting in a total of 55 samples from the five farms. Freshly dropped feces from healthy birds and intestinal contents from diseased birds that had avian colibacillosis and/or Salmonellosis were collected for analysis.

### 4.3. Study Areas and Collection of Samples

**Figure 2:**
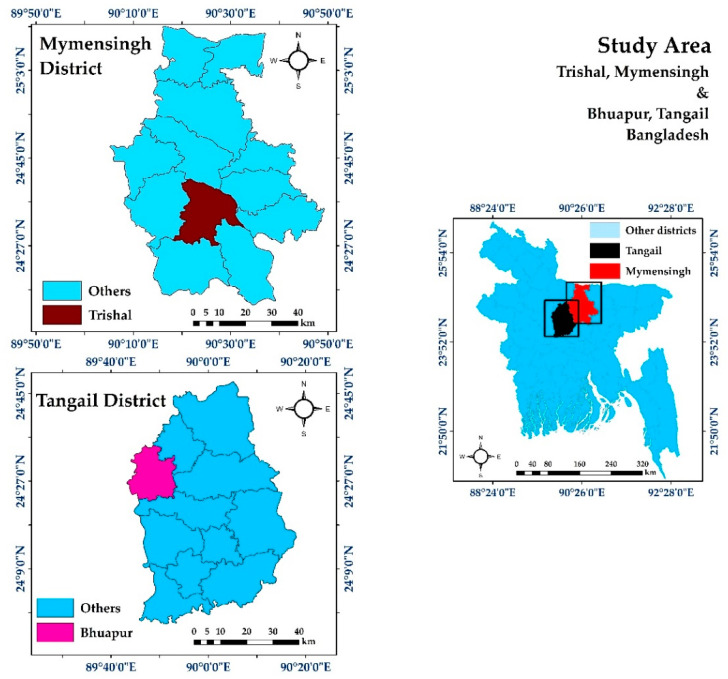
Map of the study area. Images were extracted from DIVA-GIS using Geographical Information System (GIS). The map was developed using ArcMap version 10.7.

The study was conducted in two districts of Bangladesh namely Mymensingh (24.7539° N, 90.4073° E) and Tangail (24.2513° N, 89.9167° E) during the period from June 2018 to November 2019. The study areas are represented in Figure 2.

Freshly dropped fecal samples (*n* = 30) were aseptically collected using sterile cotton buds from healthy turkeys. During the postmortem examination, 5 g of intestinal contents (*n* = 25) was collected from each turkey that died of enteritis and had lesions of avian colibacillosis and/or salmonellosis.

Immediately after collection, samples were transferred to sterile zip-lock bags. Samples were transported to the laboratory maintaining cold chain. Collected samples were transferred into sterile test tubes containing freshly prepared nutrient broth (5 mL) and were incubated aerobically at 37 °C overnight for the growth of bacteria.

### 4.4. Isolation of E. coli and Salmonella spp.

Isolation of *E. coli* and *Salmonella* spp. was based on culture on Eosin Methylene Blue (EMB) and Xylose Lysine Deoxycholate (XLD) agar (HiMedia, India) plates, respectively. Initially, freshly grown broth cultures were streaked on EMB and XLD agar media using sterile inoculating loops. This was followed by aerobic incubation of the inoculated agar plates at 37 °C overnight to obtain pure colonies. Single green-colored metallic-sheen colonies on EMB agar media and black-centered colonies on XLD agar media represented the growth of *E. coli* and *Salmonella* spp., respectively. For further confirmation, selected colonies were subjected to morphological study by Gram staining and biochemical tests such as the methyl red test, sugar fermentation test, Voges–Proskauer test, motility test, urease test, and indole test [22,31].

### 4.5. Molecular Detection of E. coli and Salmonella spp.

Isolation of *E. coli* and *Salmonella* spp. were confirmed by polymerase chain reaction (PCR) targeting *E. coli* 16S rRNA gene and *Salmonella* genus specific *invA* genes respectively (Table 3).

For PCR, genomic DNA of *E. coli* and *Salmonella* spp. was extracted by the boiling method as described by Sobur et al. [50]. Briefly, a pure colony collected from freshly grown culture was initially taken into an Eppendorf tube containing molecular-grade water (100 μL) followed by mixing gently through vortexing. Subsequently, the mixture was boiled for 10 min, cooled for 10 min, and centrifuged for 10 min at 1400 rpm. Finally, the supernatant was collected as the source for the genomic DNA for PCR and stored at −20 °C until further use.

PCR tests were carried out in a final volume of 25 μL with 12.5 μL of the master mix (2X) (Promega, Madison, WI, USA), 4 μL of genomic DNA (50 ng/μL), 1 μL of each primer, and 6.5 μL of nuclease-free water. After amplification, PCR products were subjected to gel electrophoresis in 1.5% agarose, followed by staining and visualizing by 0.25% ethidium bromide solution and ultraviolet trans-illuminator (Biometra, Göttingen, Germany). A DNA ladder (100 bp; Promega, Madison, WI, USA) was used to assess the sizes of PCR amplicons.

### 4.6. Antibiotic Sensitivity Test

Antibiotic sensitivity testing of isolated *E. coli* and *Salmonella* spp. was carried out using the disk diffusion assay as previously described [63]. Antibiotic classes included fluoroquinolones (levofloxacin, LEV—5 μg; ciprofloxacin, CIP—5 μg), aminoglycosides (gentamicin, GEN—10 μg; streptomycin, S—10 μg), carbapenems (Meropenem, MEM—10 μg; imipenem, IMP—10 μg), amphenicols (chloramphenicol, C—10 μg), macrolides (erythromycin, E—15 μg), and tetracyclines (tetracycline, TE—30 μg) purchased from Hi Media (India). Sensitivity tests were performed on freshly grown isolates having a concentration equivalent to 0.5 McFarland standard using Mueller-Hinton agar media (Hi Media, India). All results were interpreted according to the guidelines provided by Clinical and Laboratory Standards Institute [64]. Furthermore, isolates showing resistance against three or more different classes of antibiotics were defined as MDR [65].

### 4.7. Molecular Detection of Tetracycline Resistance tetA Gene

*E. coli* and *Salmonella* isolates resistant to tetracycline were screened by PCR for the detection of the tetracycline-resistance *tetA* gene using the primer and protocol described by Randall et al. [62].

### 4.8. Statistical Analysis

Chi-square tests were performed using the SPSS software (IBM SPSS version 25.0, IBM, Chicago, IL, USA). *p*-values less than 0.05 (*p* < 0.05) were considered to be statistically significant.

## 5. Conclusions

The isolation and characterization of *tetA*-gene-containing-MDR *E. coli* and *Salmonella* spp. from turkeys are concerning. The potential ability of these MDR bacteria to enter into the food chain can expose humans to serious health risks. Bacterial surveillance programs should be implemented in order to control the emergence of bacterial resistance in turkey farms in Bangladesh and elsewhere in the world. This should be a concerted effort that is best carried out via bacterial surveillance networks across different countries. Additionally, holistic and multi-sectoral approaches, such as the one health approach, need to be implemented [66]. Guided by top health professionals and scientists, these strategies can provide effective solutions to the complex, multifaceted global challenge of AMR.

## Figures and Tables

**Table 1:** Prevalence and resistance profiles of *E. coli* and *Salmonella* spp. isolated from turkeys.

Microorganism	Categories	Prevalence	Antibiotic Resistance Pattern (%)								
			LEV	E	GEN	C	CIP	S	IMP	MEM	TE
*E. coli*	Healthy	30	4	30	0	0	17	4	0	30	4
		(100)	(13.33)	(100)	(0)	(0)	(56.67)	(13.33)	(0)	(100)	(13.33)
	Diseased	25	11	25	9	11	20	5	0	10	25
		(100)	(44)	(100)	(36)	(44)	(80)	(20)	(0)	(40)	(100)
	*p*-value (Healthy vs. Diseased)	N/C	0.011	N/C	<0.001	<0.001	0.066	0.716	N/C	<0.001	<0.001
*Salmonella* spp.	Healthy	11	2	11	5	6	6	4	4	7	11
		(36.67)	(18.18)	(100)	(45.45)	(54.54)	(54.54)	(36.36)	(36.36%)	(63.63)	(100)
	Diseased	16	4	16	0	2	6	2	4	4	16
		(64)	(25)	(100)	(0)	(12.5)	(37.5)	(12.5)	(25%)	(25)	(100)
	*p*-value (Healthy vs. Diseased)	0.043	1.000	N/C	0.006	0.033	0.438	0.187	0.675	0.061	N/C

A *p*-value less than 0.05 was deemed to be statistically significant; N/C, not computed; *E. coli*, *Escherichia coli*; LEV, Levofloxacin; E, Erythromycin; GEN, Gentamicin; C, Chloramphenicol; CIP, Ciprofloxacin; S, Streptomycin; IMP, Imipenem; MEM, Meropenem; TE, Tetracycline.

**Table 2:** Multidrug resistance profiles of *E. coli* and *Salmonella* spp. isolated from healthy and diseased turkeys.

Microorganism	Source	Pattern No.	Antibiotic Resistance Patterns	No. of Antibiotics (Classes)	No. of MDR Isolates (%)	Total (%)	*p*-Value (Healthy vs. Diseased)
*E. coli* (*n* = 55)	Healthy Turkeys (*n* = 30)	1	E, MEM, CIP	3 (3)	14	24 (80%)	0.112
		2	E, MEM, TE	3 (3)	1		
		3	E, MEM, LEV	3 (3)	2		
		4	E, MEM, S	3 (3)	3		
		5	E, MEM, CIP, LEV	4 (3)	1		
		6	E, MEM, LEV, TE	4 (4)	1		
		7	E, MEM, CIP, TE	4 (4)	1		
		8	E, MEM, S, CIP, TE	5 (5)	1		
	Diseased Turkeys (*n* = 25)	1	E, CIP, TE	3 (3)	4	24 (96%)	
		2	E, MEM, TE	3 (3)	3		
		3	E, CIP, LEV, TE	4 (3)	3		
		4	E, GEN, S, CIP, TE	5 (4)	3		
		5	E, MEM, C, CIP, TE	5 (5)	2		
		6	E, MEM, C, S, TE	5 (5)	1		
		7	E, C, GEN, CIP, LEV, TE	6 (5)	4		
		8	E, MEM, C, CIP, LEV, TE	6 (5)	2		
		9	E, MEM, C, GEN, CIP, LEV, TE	7 (6)	1		
		10	E, MEM, C, GEN, S, CIP, LEV, TE	8 (6)	1		
*Salmonella* spp. (*n* = 27)	Healthy Turkeys (*n* = 11)	1	E, MEM, C, CIP, TE	5 (5)	3	8 (72.73%)	0.056
		2	E, C, GEN, CIP, TE	5 (5)	1		
		3	E, MEM, IMP, C, GEN, S, TE	7 (5)	2		
		4	E, MEM, IMP, GEN, S, CIP, LEV, TE	8 (6)	2		
	Diseased Turkeys (*n* = 16)	1	E, MEM, TE	3 (3)	3	16 (100%)	
		2	E, IMP, TE	3 (3)	3		
		3	E, CIP, TE	3 (3)	5		
		4	E, LEV, TE	3 (3)	2		
		5	E, IMP, C, TE	4 (4)	1		
		6	E, C, S, LEV, TE	5 (5)	1		
		7	E, MEM, S, CIP, LEV, TE	6 (5)	1		

A *p*-value less than 0.05 was deemed to be statistically significant; *E. coli*, *Escherichia coli*; TE, Tetracycline; E, Erythromycin; C, Chloramphenicol; LEV, Levofloxacin; GEN, Gentamicin; MEM, Meropenem; IMP, Imipenem; S, Streptomycin; CIP, Ciprofloxacin.

**Table 3:** List of primers used for detecting *E. coli*, *Salmonella* spp., and tetracycline-resistance gene.

Target Gene	Primer Sequence (5′–3′)	Amplicon Size (bp)	Annealing Temperature (°C)	References
*malB*	F: GACCTCGGTTTAGTTCACAGA R: CACACGCTGACGCTGACCA	585	55	[60]
*invA*	F: ATCAGTACCAGTCGTCTTATCTTGAT R: TCTGTTTACCGGGCATACCAT	211	58	[61]
*tetA*	F: GGTTCACTCGAACGACGTCA R: CTGTCCGACAAGTTGCATGA	577	57	[62]
